# Oncologic orphan drugs approved in the EU – do clinical trial data correspond with real-world effectiveness?

**DOI:** 10.1186/s13023-018-0900-9

**Published:** 2018-11-28

**Authors:** Yvonne Schuller, Marieke Biegstraaten, Carla E. M. Hollak, Heinz-Josef Klümpen, Christine C. Gispen-de Wied, Violeta Stoyanova-Beninska

**Affiliations:** 1 Department of Endocrinology and Metabolism, F5-165, Academic Medical Center, University of Amsterdam, Meibergdreef 9, 1105 AZ Amsterdam, The Netherlands; 2 Cancer Center Amsterdam, Academic Medical Center, University of Amsterdam, Amsterdam, the Netherlands; 3 College ter Beoordeling Geneesmiddelen/ Dutch Medicines Evaluation Board, Utrecht, the Netherlands

**Keywords:** Oncology, Orphan drugs, Efficacy, Effectiveness, Endpoints

## Abstract

**Background:**

Evaluation of evidence for efficacy of orphan medicinal products (OMPs) for rare malignancies may be hampered by the use of tumor measurements instead of clinical endpoints. This may cause efficacy data to not always match effectiveness in the real-world. We investigated whether an efficacy-effectiveness gap exists for oncologic OMPs and aimed to identify which factors contribute to it. Also, the magnitude of the clinical efficacy of oncologic OMPs was evaluated.

**Methods:**

We included all oncologic OMPs authorized in the European Union from 2000 to 2017. Pivotal studies were evaluated by means of the European Society for Medical Oncology - Magnitude of Clinical Benefit Scale (ESMO-MCBS). To estimate real-world effectiveness, a literature search was performed to identify post-marketing studies, of which data on overall survival (OS) were extracted. OS of the new OMP was compared with OS data of standard of care. An OS gain of ≥3 months compared to pre-marketing data was considered clinically relevant.

**Results:**

Twenty OMPs were included, of which 5 were authorized based on OS as a primary endpoint. 10 OMPs had post-marketing data available, of which 40% did not show a clinically relevant OS gain in the real world. All OMPs that were studied with OS as primary endpoint in the pivotal study had a clinically relevant OS gain in the real world. Furthermore, all OMPs that had a high ESMO-MCBS score and post-marketing data available, resulted in a clinically relevant OS gain in the real world.

**Conclusions:**

Although the sample size is small, our results indicate an efficacy-effectiveness gap for oncologic OMPs exists. Significant changes in PFS do not always lead to an increased OS. The use of PFS may be justified, but validation of surrogate endpoints is needed.

**Electronic supplementary material:**

The online version of this article (10.1186/s13023-018-0900-9) contains supplementary material, which is available to authorized users.

## Background

Cancer accounts for 14 million new cases and over 8 million cancer related deaths worldwide per year [1]. Since the cancer burden is expected to rise to 22 million new cases annually within the next two decades [2], there is a need for the development of new, effective oncologic drugs that can reach patients timely. However, in the case of rare cancers, the small consumer’s market offered little attraction to the pharmaceutical industry. To stimulate development of drugs for rare (‘orphan’) diseases, orphan drug (‘orphan medicinal product’, OMP) legislation was enacted in the EU in 2000 (Table 1). This legislation is similar but not identical to orphan drug legislation in the United States or Japan, and consists of several incentives, such as 10 years of market exclusivity after authorization of the drug, and reduction of registration costs. Stimulating orphan drug development by introducing incentives has led to a significant increase in the amount of OMPs. Although assessment of efficacy for OMPs and non-OMPs are generally similar, differences may exist with regard to clinically relevant endpoints used [3]. Tumor measurements (Table 2) that are not validated as surrogate endpoints for overall survival (OS) are being used with increasing frequency over the past years, while the use of OS as a primary endpoint is declining. Although both the United States Food and Drug Administration (FDA) and the European Medicines Agency (EMA) agreed that OS is the most reliable and persuasive outcome [4, 5], progression free survival (PFS) is broadly used in studies. Using tumor measurements instead of clinical endpoints requires a smaller trial population and a shorter follow-up to show statistical significant evidence on efficacy. However, the drawback of allowing tumor measurements to be the basis for approval of OMPs, is that efficacy data do not always match real-world effectiveness, a phenomenon that is called the ‘efficacy-effectiveness gap’. Literature shows that, for example, PFS, is not always a good surrogate for OS [6]. Also, a previous study on oncologic OMPs approved by the FDA showed that two-thirds of the approvals were based on an improvement on a surrogate endpoint (such as PFS) [7]. Of these, 86% had unknown effects on OS or failed to show gains in survival after several years of follow-up [7]. Based on this, it may be discussed whether the use of PFS as an endpoint is preferable or not. Finally, randomized controlled trials are often performed on a homogenous population, from which patients with comorbidities are generally excluded for the sake of study feasibility [8]. Through the strict in- and exclusion criteria, the external validity of trials may be called into question; patients treated in routine practice may have shorter survival and more toxicity than patients treated in the context of a clinical trial [9, 10].

**Table 1:** Orphan diseases and OMPs

The European Commission is responsible for authorizing OMPs in the European Union (EU), after careful evaluation of the OMP’s benefits and risks by the European Medicines Agency. An OMP is a drug that is indicated for the treatment of a rare disease with a prevalence of < 5:10000. A centralized procedure allows applicants to obtain a marketing authorization that is valid throughout the EU as well as in the European Economic Area, and is compulsory for the authorization of OMPs and medicines containing a new active substance to treat cancer [64]. Marketing authorizations may be ‘full’ (sufficient comprehensive data are available), ‘conditional’ (benefit of immediate authorization outweighs the risk of less comprehensive data than normally required, but additional data are expected to be generated in the future) or ‘exceptional’ (authorization is granted even though comprehensive data are not expected to be obtained after authorization) [65].

**Table 2:** Definitions of endpoints [4, 5]

Although OS seems to be the most reliable outcome, other frequently used outcomes include tumor measurements and biomarkers. PFS or DFS may be considered to be of benefit to the patient, but they are often not validated as surrogate outcomes for OS. The validation of tumor measurements and biomarkers as surrogates for survival is difficult. For each indication separately, robust evaluations are needed to investigate whether a correlation exists between effects on survival and tumor measurements or biomarkers. For the sake of clarity, categories of endpoints as used in this manuscript are stated below. 1. Overall survival (OS): Time from randomization until death from any cause. 2. Tumor measurements: Progression-free survival (PFS), disease-free survival (DFS) (=recurrence-free survival (RFS)), time to progression (TTP), time to treatment failure (TTF), objective response rate (ORR). 3. Symptom assessment: Time to progression of cancer symptoms, quality of life (QoL) 4. Biomarker: Characteristic that should be capable of objectively measuring and evaluating a normal biological process, a pathological process or the pharmacological response to a therapeutic intervention. Generally assayed from blood or body fluids. The validity of biomarkers as endpoints in trials remains to be established.	

For this study, we aimed to explore whether there is a difference between efficacy and effectiveness of oncologic OMPs approved in the EU and to evaluate which factors contribute to the efficacy-effectiveness gap. We herewith aim to provide possible solutions for regulators, academia and industry on how to bridge this gap. Although similar studies were performed for oncology drugs approved by the EMA and FDA, these did not focus on orphan drugs and did not always include post-marketing data [7, 11, 12]. In light of the increasing discussion about orphan drugs and their prices, this study could be of high societal value. Furthermore, in this study we additionally aimed to rank the magnitude of the clinical efficacy of oncologic OMPs at the time of marketing authorization by using the European Society for Medical Oncology - Magnitude of Clinical Benefit Scale (ESMO-MCBS).

## Methods

### Efficacy

All oncologic OMPs that were authorized by the European Commission in the EU from the implementation of EU orphan drug legislation in 2000 until January 1st 2017 were included in our study, by consultation of the ‘Community register of orphan medicinal products for human use’ of the European Commission. OMPs that were withdrawn from the EU market or removed from the community register at the end of the 10-year market exclusivity were also included. Both initial marketing authorizations (the first disease for which an OMP was marketed) and extensions (subsequent diseases for which authorization was extended) were included. OMPs that were designated ‘orphan status’, but had not (yet) received EMA marketing authorization, were not included. All the pivotal studies that led to marketing authorization of oncological OMPs were evaluated by means of ‘COMPASS’ (Clinical evidence of Orphan Medicinal Products - an ASSessment tool) [13]. This tool was developed to assess the quality of OMP’s clinical evidence and mainly focuses on study design and methods. It was not developed to score the quality of clinical evidence, but rather to provide guidance on assessing the value of clinical evidence [14]. One author (YS) completed all COMPASS assessments. If uncertainty existed, another author (MB) was consulted, with whom the questions were discussed until consensus was achieved [14].

Whether the study population was a reliable reflection of the patient population, was evaluated based on Eastern Cooperative Oncology Group (ECOG) performance status. In case a Karnofsky status was used, this was converted into ECOG performance status according to the following categories: Karnofsky 90-100 = ECOG 0, Karnofsky 70-80 = ECOG 1, Karnofsky 50-60 = ECOG 2, Karnofsky 30-40 = ECOG 3 [15]. A study population that consisted only of patients with ECOG performance status 0-1 was considered not to be a reliable reflection of the total patient population.

Additionally, the ESMO-MCBS was used to rank the magnitude of the clinical efficacy of the oncologic OMPs that were marketed [16]. ESMO-MCBS can be used to assess drugs for the treatment of solid cancers, and it can only be applied to either randomized or comparative cohort studies evaluating the relative benefit of treatments using outcomes of survival, quality of life (QoL), surrogate outcomes for survival or QoL, or treatment toxicity. Different forms are used to evaluate drugs: [16] Form 1 is used for curative (neo)adjuvant therapies and uses a scale with grades A, B, or C, of which grades A and B represent a high level of clinical benefit. Form 2 is divided into 3 sub forms and is used for palliative interventions. Form 2a is used for studies with OS as primary outcome, form 2b for studies with PFS or time to progression (TTP) as primary outcome, and 2c for studies with QoL, toxicity or objective response rate (ORR) as primary outcome as well as for non-inferiority studies. The scale in form 2 is graded 1-5, where grades 5 and 4 represent a high level of clinical benefit [16]. All pivotal studies were scored by one researcher (YS). In case of doubt, a second researcher (MB) was consulted.

### Effectiveness

For each oncologic OMP included in our study, a review of the literature was conducted in the Medline database in January 2017 to search for post-marketing studies. Search terms included the OMP’s generic and trade name, disease name and alternative notations. Randomized controlled trials, observational studies, and, if these were not available, case series were included, as were follow-up data of pivotal studies that were published after marketing authorization. Articles were only included if the OMP was used according to the authorized indication. Studies for which no full text was available were excluded from our analysis, as were studies that only evaluated a subset of the patients of the pivotal study. Point estimates of OS data of standard of care were obtained from European Public Assessment Reports (EPARs) or the introduction section of the pivotal study. If not available, scientific literature was consulted. Point estimates of OS data were extracted from all post-marketing studies. In order to analyze the magnitude of OS gain, the lowest point estimate of post-marketing OS was compared to the highest point estimate of pre-marketing OS data from standard of care (before the OMP was authorized). This was considered to be the most appropriate approach to identify a reliable OS difference. Subsequently, the real-world effectiveness of each OMP was categorized as follows: 1) no demonstrated increase in OS, 2) gain in OS, but of unknown magnitude, 3) gain in OS of < 3 months, 4) gain in OS of ≥3 months [17]. We considered an OS gain of ≥3 months as clinically relevant, which is in line with both the National Institute for Health and Care Excellence (NICE) and the Dutch Committee of Oncologic Drugs [18]. The former generated advice on ‘appraising life-extending, end of life treatments’, to be taken into account when appraising treatments which may be life-extending for patients with short life-expectancy. The latter gives a positive advice only when the (progression-free) survival gain is 12 weeks. This advice does not take into account the prevalence of the indication.

### Data analysis

For the purpose of the analyses, the real-world effectiveness was dichotomized into ‘no or unclear effect’ (no OS gain, OS gain of unknown magnitude or OS gain < 3 months) or ‘good effect’ (OS gain ≥3 months). To assess the presence of an efficacy-effectiveness gap, the percentage of OMPs that had ‘no or unclear effect’ in the real-world was calculated. COMPASS variables (i.e. type of primary endpoint, performance status, type of marketing authorization, study phase, study power, early study termination and randomization) were used to investigate which determinants were associated with a ‘good effect’ in the real-world setting. In addition, we evaluated whether the ESMO-MCBS can be used to predict OS gain in the real world. For this analysis, ESMO-MCBS scores were dichotomized into ‘clinically beneficial’ (grades A/B (form 1), or 4/5 (form 2)) or ‘not beneficial’ (grade C (form 1), or 3/2/1 (form 2)). These scores were subsequently compared to the effect in the real world (i.e. ‘no or unclear effect’ or ‘good effect’). Descriptive statistics were used due to the small number of OMPs included. All analyses were performed using IBM SPSS Statistics version 22.

## Results

Table 3 represents an overview of all OMPs that were marketed from 2000 until the 1st of January 2017. Sixteen OMPs were marketed for 15 indications, based on 24 pivotal studies. One OMP (Gliolan®, 5-aminolevulinic acid hydrochloride) was excluded from the analyses, since it is used as a tool aimed at improving surgical resection of gliomas. Since some OMPs were authorized for more than one indication, they were counted twice or thrice. The results presented here hence include a total of 20 OMPs for 14 indications, based on 23 pivotal studies.

**Table 3:** Overview of included OMPs

Drug	Generic name	Year of authorization	Type of authorization	Disease	Specification of indication
Glivec^a^	imatinib	2002 2006	Exceptional	1. GIST 2. DFSP	1. Unresectable or metastatic GISTs. 2. Unresectable or metastatic DFSP, and adults who are not eligible for surgery after disease recurrence.
Lysodren^a^	mitotane	2004	Full	Adrenocortical carcinoma	Unresectable, metastatic or relapsed adrenocortical carcinoma.
Sutent^a^	sunitinib	2006 2006	Conditional	1. GIST 2. RCC	1. Unresectable or metastatic GISTs. Used after treatment with imatinib has failed. 2. Metastatic RCC. Patients who failed to respond to or relapsed after IFN-a or IL-2 based therapy.
Nexavar^a^	sorafenib	2006 2007 2014	Full	1. RCC 2. HCC 3. Differentiated thyroid carcinoma	1. Advanced RCC. Patients who have failed to prior IFN-a or IL-2 based therapy. 2. Not specified. 3. Locally advanced or metastatic differentiated thyroid carcinoma refractory to RAI.
Torisel	temsirolimus	2007	Full	RCC	Advanced RCC. Patients who have not previously received systemic therapy and who have at least 3 of 6 prognostic risk factors.
Yondelis	trabectedine	2007 2009	Exceptional	1. STS 2. Ovarian cancer	1. Advanced STS, patients in which anthracyclines and ifosfamide failed, or who are unsuited to receive these agents. 2. Relapsed ovarian cancer, sensitive to platinum-based chemotherapy. Used in combination with pegylated liposomal doxorubicin.
Mepact	mifamurtide	2009	Full	Osteosarcoma	High-grade resectable non-metastatic osteosarcoma after macroscopically complete surgical resection. Combined with post-operative multi-agent chemotherapy.
Afinitor^a^	everolimus	2009	Full	RCC	Advanced RCC when relapsed after treatment with a VEGF-targeted medicine.
Cometriq	cabozantinib	2014	Conditional	Medullary thyroid cancer	Progressive, unresectable locally advanced or metastatic medullary thyroid carcinoma.
Lynparza	olaparib	2014	Full	Ovarian cancer	Maintenance therapy of adult patients with platinum-sensitive relapsed BRCA-mutated ovarian cancer who are in response to platinum-based chemotherapy.
Cyramza^a^	ramucirumab	2014	Full	Gastric cancer	Advanced gastric cancer or gastro-oesophageal junction adenocarcinoma with disease progression after prior platinum and fluoropyrimidine chemotherapy. Combined with paclitaxel.
Unituxin^b^	dinutuximab	2015	Full	Neuroblastoma	Patients aged 11 months to 17 years with high-risk neuroblastoma, following myeloablative therapy and ASCT, in combination with GM-CSF, IL-2 and isotretinoin.
Lenvima	lenvatinib	2015	Full	Differentiated thyroid carcinoma	Progressive, locally advanced or metastatic differentiated (papillary/follicular/Hürthle cell) thyroid carcinoma, refractory to RAI.
Lartruvo	olaratumab	2016	Conditional	STS	Adult patients with advanced STS, not amenable to curative treatment with surgery or radiotherapy and not previously treated with doxorubicin. Used in combination with doxorubicin.
Onivyde	nanoliposomal irinotecan	2016	Full	Pancreatic cancer	Metastatic adenocarcinoma of the pancreas, in combination with 5-FU and LV, in adult patients who have progressed following gemcitabine based therapy.

*5-FU* 5 fluorouracil, *ASCT* autologous stem cell transplant, *BRCA* breast cancer, *DB* double-blind, *DFSP* dermatofibrosarcoma protuberans, *EFS* event-free survival, *GIST* gastrointestinal stromal tumor, *GM-CSF* granulocyte-macrophage colony-stimulating factor, *HCC* hepatocellular carcinoma, *Her2Neu* Human Epidermal growth factor Receptor 2, *HR* hormone receptor, *IFN-a* interferon-alpha, *IL-2* interleukin-2, *LV* leucovorin, *OMP* orphan medicinal product, *OS* overall survival, *ORR* objective response rate, *Pbo* placebo, *PFS* progression-free survival, *pNET* pancreatic neuroendocrine tumor, *RAI* radioactive iodine, *RCC* renal cell carcinoma, *RCT* randomized controlled trial, *RFS* recurrence-free survival, *SAT* single-arm trial, *STS* soft tissue sarcoma, *TTP* time to progression, *VEGF* vascular endothelial growth factor

^a^No longer an orphan drug. Withdrawn from the Community register of OMPs upon request of the marketing authorisation holder or after 10 years of market exclusivity.

^b^Withdrawn from use in the European Union at the request of the marketing authorisation holder due to short- and intermediate- term inability to supply Unituxin in sufficient quantities for meeting current global demands

All information in this table was retrieved from http://www.ema.europa.eu/ema/

### Efficacy - COMPASS tool

Table 4 shows the most important findings of the COMPASS tool. A mean of 431 patients were included in the pivotal studies. The majority of the studies was phase III (16/23, 70%) [19–34] and randomized (20/23, 90%) [19–38]. Of the randomized studies, 11/20 (55%) [20–25, 27, 30, 32, 34, 37] were double-blind. One OMP (Lysodren®, mitotane) was authorized based on ‘well-established-use’, hence multiple studies and endpoints were used. Six of the pivotal studies (6/23, 26%) [24–27, 33, 34] corresponding with 5 OMPs (5/20, 25%) used OS as a primary endpoint. Two studies (2/23, 9%) [29, 37] corresponding with 2 OMPs (2/20, 10%) used a surrogate endpoint for OS (i.e. PFS for ovarian cancer). In the remaining 14 pivotal studies [19–23, 28, 30–32, 35, 36, 38–40] tumor measurements were used as primary endpoint. Only 1/14 pivotal studies (7%) [38] that used a tumor measurement as primary endpoint also showed a statistically significant improvement in OS (measured as secondary endpoint). In the other studies, OS was either not analyzed, or not yet reached, or did not improve significantly. Most studies (21/23, 91%) [19–27, 29–40] included a minimum performance status (either ECOG or Karnofsky) in their eligibility criteria. Nine studies (9/23, 39%) [22, 24, 26, 30, 31, 33–35, 40] only included patients with ECOG status 0-1, indicating a selected study population consisting of patients who are ambulatory and able to carry out work of a light or sedentary nature [15]. Eleven studies (11/23, 48%) [20, 24–26, 29–31, 33, 34, 37, 39] included QoL as secondary endpoint, of which only 1 (1/23, 4%) [31] study showed a statistically significant improvement. Four studies (4/23, 17%) [19, 22, 24, 30] were terminated early, mostly because interim analyses showed superiority of the new OMP over placebo (Additional file 1).

**Table 4:** Characteristics of pivotal studies (COMPASS)

Drug	Indication	Pivotal study	Primary endpoint	Study phase	Control	Design	Performance status [16]	No. of patients	OS effect in months	QoL effect
Imatinib	GIST	Demetri 2002 [36] DeMatteo 2009 [21]	ORR RFS	II III	Imatinib (different dose) Placebo	Rand. OL Rand. DB	ECOG ≤3 ECOG ≤2	147 713	n/a n/a	NE NE
Imatinib	DFSP	Heinrich 2008 [39]	ORR	II	None	Single-arm	ECOG ≤2	18	NE	NS
Mitotane	Adrenocortical carcinoma	Several pivotal studies	OS, PFS, ORR	n/a	n/a	n/a	n/a	> 500	n/a	n/a
Sunitinib^a^	GIST	Demetri 2006 [22]	TTP	III	Placebo	Rand. DB	ECOG ≤1	312	n/a	NE
Sunitinib	RCC	Motzer 2006 [40] Motzer 2007 [31]	ORR PFS	II III	None IFN-α	Single-arm Rand. OL	ECOG ≤1 ECOG ≤1	106 750	n/a n/a	NE Improved
Sorafenib^a^	RCC	Escudier 2007 [24]	OS	III	Placebo	Rand. DB	ECOG ≤1	903	n/a	NS
Sorafenib	HCC	Llovet 2008 29	OS, TTP	III	Placebo	Rand. DB	ECOG ≤2	602	2.8	NE
Sorafenib	Thyroid carcinoma	Brose 2014 [20] (*DECISION*)	PFS	III	Placebo	Rand. DB	ECOG ≤2	417	n/a	NS
Temsirolimus	RCC	Hudes 2007 [26] (*ARCC*)	OS	III	IFN-α	Rand. OL	Karnofsky ≥60 (ECOG 1)	626	3.6	NS
Trabectedin	STS	Demetri 2009 [35]	TTP	II	Trabectedin (different dose)	Rand. OL	ECOG ≤1	266	2.1 (NS)	NE
Trabectedin	Ovarian cancer	Monk 2010 [29]	PFS	III	Single-agent PLD	Rand. OL	ECOG ≤2	672	n/a	NS
Mifamurtide	Osteosarcoma	Meyers 2005 [28]	EFS	III	Ifosfamide	Rand. OL (factorial)	Not reported	678	n/a	NE
Everolimus^a^	RCC	Motzer 2008 [30]	PFS	III	Placebo	Rand. DB	Karnofsky ≥70 (ECOG 1)	416	n/a	NS
Cabozantinib	Medullary thyroid cancer	Elisei 2013 [23]	PFS	III	Placebo	Rand. DB	ECOG ≤2	330	n/a	NE
Olaparib	Ovarian cancer	Ledermann 2012 [37]	PFS	II	Placebo	Rand. DB	ECOG ≤2	265	n/a	NS
Ramucirumab	Gastric cancer	Wilke 2014 [34] (*RAINBOW*) Fuchs 2014 [25] (*REGARD*)	OS OS	III III	Placebo Placebo	Rand. DB Rand. DB	ECOG ≤1 ECOG ≤2	665 355	2.2 1.4	NS NS
Dinutuximab^a^	Neuroblastoma	Study not yet published [19]	EFS	III	Isotretinoin	Rand. OL	Karnofsky ≥50 (ECOG 2)	230	n/a	NE
Lenvatinib	Thyroid cancer	Schlumberger 2015 [32]	PFS	III	Placebo	Rand. DB	ECOG ≤3	392	n/a	NE
Olaratumab	STS	Tap 2016 [38]	PFS	Ib/II	Doxorubicin	Rand. OL	ECOG ≤2	133	11.8	NE
Nanoliposomal irinotecan	Pancreatic cancer	Wang-Gillam 2016 [33]	OS	III	Fluorouracil and folinic acid	Rand. OL	Karnofsky ≥70(ECOG 1)	417	1.9	NS

*COMPASS* Clinical Evidence of Orphan Medicinal Products – an ASSessment tool, *DB* double-blind*, DFSP* Dermatofibrosarcoma protuberans*, ECOG* Eastern Cooperative Oncology Group, *EFS* event-free survival, *GIST* gastrointestinal stromal tumor, *HCC* hepatocellular carcinoma, *IFN-α* interferon alfa, *NE* not evaluated, *NS* no significant improvement, *ORR* overall response rate, *OS* overall survival, *PFS* progression-free survival, *PLD* pegylated liposomal doxorubicin, *pNET* pancreatic neuroendocrine tumor, *PLD* pegylated liposomal doxorubicin, *Rand DB* randomized double-blind, *Rand OL* randomized open-label, *RCC* renal cell carcinoma, *STS* soft tissue sarcoma, *TTP* time to tumor progression

^a^ Pivotal study was terminated early

### Efficacy - ESMO-MCBS

Table 5 shows the ESMO-MCBS results of the pivotal studies. For three indications, two pivotal studies were used as a basis for marketing authorization. In these cases the study with the highest ESMO-MCBS score prevailed, resulting in a total of 20 pivotal studies. Five out of 20 studies (25%) [19, 21, 26, 31, 38] scored a grade A/B (form 1), or 5/4 (form 2), representing a high level of proven clinical benefit [16]. The other pivotal studies (15/20, 75%) [20, 22–24, 27–30, 32–35, 37, 39] scored C or ≤ 3, meaning that the level of clinical benefit was uncertain at time of marketing authorization.

**Table 5:** ESMO-MCBS scores and comparison of OS before marketing authorization and after marketing authorization

Drug	Indication	Score ESMO-MCBS	Median OS of standard of care (before authorization)	Median OS of OMP (after authorization)	OS gain (before authorization vs after authorization)
Imatinib	GIST	A	9 – 22 months	41.1 months	≥3 months
Imatinib	DFSP	n.a.	3-year OS rate 66%	3-year OS rate 60 – 77%	No gain
Mitotane	Adrenocortical carcinoma	n.a.	3 – 9 months	No post-marketing studies	n.a.
Sunitinib	GIST	3	9 – 22 months	14.1 – 17.6 months	Gain of unknown magnitude
Sunitinib	RCC	4	10 – 13 months	18.2 – 27.2 months	≥3 months
Sorafenib	RCC	3	10 – 13 months	17.8 – 29.3 months	≥3 months
Sorafenib	HCC	3	< 6 months	5 – 10.2 months	≥3 months
Sorafenib	Thyroid carcinoma	2	2.5 – 3.5 years	2.4 – 4.7 years	Gain of unknown magnitude
Temsirolimus	RCC	4	6 – 12 months	11.6 – 18 months	≥3 months
Trabectedin	STS	1	6 months	11.9 – 19.3 months	≥3 months
Trabectedin	Ovarian cancer	2	> 30 months	16.3 – 22.2 months	No gain
Mifamurtide	Osteosarcoma	C	13 – 28 months	No post-marketing studies	n.a.
Everolimus	RCC	2	5-year survival rate 9.5%	14.8 – 32 months	n.a.
Cabozantinib	Medullary thyroid cancer	3	10-year survival rate < 40%	No post-marketing studies	n.a.
Olaparib	Ovarian cancer	2	5-year OS 44% for BRCA1 carriers, 52% for BRCA2 carriers	No post-marketing studies	n.a.
Ramucirumab	Gastric cancer	2	12 months	No post-marketing studies	n.a.
Dinutuximab	Neuroblastoma	A0	EFS rates are 30 - 40% for children with high-risk neuroblastoma	No post-marketing studies	n.a.
Lenvatinib	Thyroid cancer	2	2.5 – 3.5 years	No post-marketing studies	n.a.
Olaratumab	STS	4	11 – 15 months	No post-marketing studies	n.a.
Nanoliposomal irinotecan	Pancreatic cancer	2	< 12 months	No post-marketing studies	n.a.

*BRCA* breast cancer, *DFSP* dermatofibrosarcoma protuberans, *EFS* event-free survival, *ESMO-MCBS* European Society for Medical Oncology - Magnitude of Clinical Benefit Scale, *GIST* gastrointestinal stromal tumor, *HCC* hepatocellular carcinoma, *OMP* orphan medicinal product, *OS* overall survival, *pNET* pancreatic neuroendocrine tumor, *RCC* renal cell carcinoma, *STS* soft tissue sarcoma

### Effectiveness – OS gain in the real-world setting

For the analysis of real-world effectiveness and the efficacy-effectiveness gap, ten OMPs were excluded from our analysis, since either no post-marketing studies were performed, or OS gain could not be determined due to a lack of standard of care data. Of the remaining 10 OMPs, 59 post-marketing studies were identified. Table 5 and Additional file 2 provide an overview of these studies and their results.

### Efficacy-effectiveness gap

For 2 OMPs (2/10, 20%) no OS gain in the real world setting was found, 2 OMPs (2/10, 20%) had OS gains of unknown magnitude and none had OS gains of < 3 months, making a total of 4 OMPs (4/10, 40%) with ‘no or unclear effect’ in the real-world, and 6 out of 10 OMPs (60%) with a ‘good effect’ (OS gain ≥3 months) (Table 5).

### Determinants of a clinically relevant gain in OS in the real world

COMPASS variables and ESMO-MCBS scores were used to determine which factors predict a ‘good effect’ (OS gain ≥3 months) in the real world. Results are shown in Tables 6, 7, 8 and 9. With respect to the COMPASS variables, all 3 OMPs that used OS as primary endpoint in the pivotal study, had a ‘good effect’ in the real world setting. The OMP that used a surrogate for OS in the pivotal study, showed no OS gain in the real world. Contrarily, of the 6 OMPs for which a tumor measurement (PFS, RFS, TTP, ORR) was used as primary endpoint in the pivotal study, 3 (3/6, 50%) had a ‘good effect’ in the real world. OMPs that were granted a full marketing authorization were effective in 3 of the 4 cases (75%). This is higher than OMPs that were granted a conditional or exceptional marketing authorization, where only 3/6 (50%) showed a good real-world effectiveness. None of the 2 OMPs that were authorized based upon underpowered studies showed a good effect post-marketing, while 75% of the well-powered studies showed a good effect. Although differences were observed, none of the above described associations showed statistical significance in the Fisher’s exact test. The other COMPASS variables (performance status, study phase, randomization and early study termination) did not show a relevant association with an OS gain of ≥3 months in the real-world setting (percentage difference ≤ 25%, data not shown).

**Table 6:** Relation between ESMO-MCBS score and real-world effectiveness

		Effectiveness		
		Survival benefit > 3 months	Survival benefit < 3 months, unknown magnitude or no benefit	Total
ESMO-score	Score A, B, 5 or 4	3	0	3
	Score C, 1, 2 or 3	3	4	7
	Total	6	4	10

**Table 7:** Relation between type of primary endpoint and effectiveness

		Effectiveness		
		Survival benefit > 3 months	Survival benefit < 3 months, unknown magnitude or no benefit	Total
Primary endpoint	OS or surrogate endpoint for OS	3	1	4
	PFS, RFS, TTP, ORR	3	3	6
	Total	6	4	10

**Table 8:** Relation between type of authorization and effectiveness

		Effectiveness		
		Survival benefit > 3 months	Survival benefit < 3 months, unknown magnitude or no benefit	Total
Authorization	Full	3	1	4
	Conditional or exceptional	3	3	6
	Total	6	4	10

**Table 9:** Relation between study power and effectiveness

		Effectiveness		
		Survival benefit > 3 months	Survival benefit < 3 months, unknown magnitude or no benefit	Total
Study power	Adequately powered	6	2	8
	Underpowered	0	2	2
	Total	6	4	10

All OMPs that were considered ‘clinically beneficial’ according to the ESMO-MCBS showed a ‘good effect’ post-marketing, while only 3 of the 7 OMPs (43%) that were ‘not beneficial’ according to the ESMO-MCBS showed a ‘good effect’ (Table 6).

## Discussion

We aimed to investigate the presence of an efficacy-effectiveness gap of oncologic OMPs authorized in the EU, and its contributing factors. Despite the small sample size, some general conclusions can be drawn from our data: 40% of the OMPs included in our analysis did not show a clinically relevant gain in OS in the real-world setting, and only 25% of the OMPs had a high level of proven clinical benefit at time of marketing authorization according to the ESMO-MCBS scale. This illustrates that the extent to which results from pivotal trials translate into the general patient population is uncertain, which has also been shown by another study [7]. While this previous study focused on cancer drugs approved by the FDA on the basis of a surrogate endpoint, our study concentrates on orphan oncology drugs approved by the EMA.

### Choosing the right endpoint

Our study showed that half of the oncologic OMPs that were approved on the basis of an effect on a tumor measurement did not show a significant improvement in OS. The use of endpoints other than OS in clinical studies remains an important matter of discussion, since improvements in PFS do not always translate into a similar improvement in OS in the real-world setting. This discrepancy between PFS and OS may be explained by several factors, including the finding that some drugs can delay progression, but can also lead to changes in tumors, producing a more aggressive phenotype after treatment [6].

Using PFS as a surrogate for OS is only justifiable if it is validated as a surrogate endpoint. To date, hepatocellular carcinoma, advanced colon carcinoma and ovarian cancer are the only indications for which strong validation evidence is available [41, 42]. For other tumor types (e.g. breast cancer) PFS has been suggested to be a suitable surrogate endpoint, but further evidence is needed to support this [41]. To validate a surrogate endpoint, a meta-analysis has to be performed, in which evidence from several studies that measure OS without confounding is combined [41]. Thus, the use of PFS is to be discouraged unless validation studies have demonstrated that it translates into a better OS for the disease under study [43]. If this is considered unfeasible given the rare nature of the indication, post-marketing obligations are needed to encourage the marketing authorization holder to demonstrate OS gains after marketing authorization. This is also discussed below.

But even the use of OS has its limitations: the practice of crossover after disease progression and the confounding effect of 2nd and 3rd line treatment options that are administered after disease recurrence which may occur during or after study termination may hamper the determination of OS. Also, in slowly progressive cancers (e.g. gastrointestinal stromal tumor, neuroendocrine tumors), OS may not be the most realistic and appropriate primary endpoint. In these cases, the use of a (validated) surrogate endpoint may be the second best option [44]. These considerations are strictly related to the specific oncological condition. In view of the above, alternative endpoints should be considered.

### Quality of life and toxicity

Over the past years, the social debate has focused increasingly on QoL of cancer patients, instead of just improvements in OS. QoL or toxicity, which are likely to be related to each other [5], therefore seem to be attractive alternatives to improved OS. Although QoL as the only outcome measure of effectiveness might not be desirable, evaluating QoL and toxicity in combination with survival measures could be of great value. Especially when OS effects are only small, it is important to place them in context with treatment toxicity and QoL [45]. A longer PFS with a good QoL may be considered as clinically relevant despite a lack of effect on OS. Proof of an association between PFS and QoL is still scarce; while some studies report weak or insufficient evidence of an association, others show that tumor progression in patients with lung cancer is associated with statistically significant worsening in QoL [46–48].

For tumor types for which PFS is not yet a validated surrogate for OS, it is interesting to investigate whether the treatment under investigation does lead to an improved QoL or prevent serious deterioration in QoL. In this regard, it is remarkable that QoL data are inadequately reported in the majority of the studies included in our analysis. Earlier studies have also shown that QoL has generally not been well reported in clinical trials [49, 50]. Although toxicity is compulsorily reported in registration dossiers and generally reported in studies, this is usually not done in relation to QoL [51]. Future studies should therefore focus on measuring QoL, also in relation to toxicity, using appropriate patient-reported outcome measures.

### Post-marketing follow-up

The results from our research indicate that OMPs that are granted a conditional or exceptional marketing authorization are less often effective in the real-world (i.e. an OS gain ≥3 months) than OMPs that were granted a full marketing authorization. It is therefore important that post-marketing studies (clinical trials, observational studies or disease registries) of sufficient quality and in a broader patient population are performed in case of conditional or exceptional approval. However, research has shown that two thirds of the post-marketing studies reported results after their original FDA report submission deadline, and the results were often briefly described and difficult to categorize [52]. Although compliance of conducting such studies by marketing authorization holders in the EU was never thoroughly investigated, it is known that half of the post-marketing studies attached to a conditional marketing are completed with a substantial delay [7, 53–56]. In the past, some OMPs were withdrawn from the market due to serious safety concerns [57], but it seems more problematic to remove an OMP from the market due to questionable efficacy in general and lack of an improvement in OS more specifically in the post-marketing setting [49]. In order to establish what represents sufficient evidence at time of marketing authorization and in the post-marketing period, it is important to involve regulators and industry, but also academics and patients early in the drug development process. Approving an OMP based on an effect on a tumor measurement may be acceptable if strict regulations require data on clinical outcomes in the post-marketing phase. Also, regarding the difficulties involving OMP development, it has to be agreed upon in an early phase what constitutes a suitable study design.

Several databases exist with publicly available data on survival associated by age, sex, race, year of diagnosis, and geographic area (Netherlands Comprehensive Cancer Organization and the Surveillance, Epidemiology, and End Results Program). However, these databases do not provide information on the previous treatments and their outcome for each patient included in the database [58]. This information would especially be of importance in OMPs that are authorized to be used as a 2nd or 3rd line therapy. Also, a priori defined analysis of OS data in patient subgroups could provide additional important information. Therefore, initiatives such as the National Cancer Knowledge System, which is part of the US Precision Medicine Initiative® and which integrates genomic information from tumors with clinical response data, should be highly encouraged [59].

### Bridging the efficacy-effectiveness gap: Future recommendations

As shown by the ESMO-MCBS scores, all OMPs of which the pivotal studies showed a high level of clinical benefit (scores A, B, 5 or 4), are effective in the real world. This finding suggests that the ESMO-MCBS results may be used by the regulators in the assessment of clinical trial data, but also by others in clinical guideline development and health technology assessment (HTA)-analyses. Although ESMO-MCBS takes into account QoL and toxicity, these outcomes are not always reported well in the literature. Therefore, future studies should put greater emphasis on measuring and reporting QoL and toxicity.

Signals for the need of a better alignment between the data requirements of both the regulators and HTA bodies, with the aim of bridging or at least reducing the efficacy-effectiveness gap, have resulted in several recent initiatives by the EMA in the past years. First, a pilot was started on a novel way of marketing authorization, the so-called ‘adaptive pathway’. This approach addresses the issue of subpopulations in orphan diseases. A marketing authorization is first granted to a small, well-defined subpopulation in which the drug is proven to be effective. With additional evidence, the target population may be broadened [60]. As long as it is unclear whether a new OMP is effective in a broader population, strict prescription rules should be applied. The idea is that the HTA decisions are taken stepwise accordingly. In another initiative, called PRIority MEdicines (PRIME), early dialogue with HTAs is less prominent. It does, however, offer early support to the pharmaceutical industry to optimize data generation and enables accelerated assessment of medicines that may offer a major therapeutic advantage over existing treatments, or a benefit to patients with no treatment options [61]. Up to July 2017, 6 oncological medicines are enrolled in a PRIME scheme. Another important development by the EMA is the ‘real-world evidence’ initiative, which aims to collect data on effectiveness outside the constraints of conventional randomized clinical trials (e.g., biobanks, insurance data, registries) [62]. By collecting real-world evidence throughout the lifecycle of a drug, decision-making for regulators and HTA bodies will eventually be improved. For post-marketing OMP registries, criteria have been proposed to improve their quality and value [63].

### Limitations

The most important limitation of the current study is the small sample size, which hampered the performance of statistical analyses. Furthermore, post-marketing OS data were only available for half of the OMPs, which made it challenging to draw conclusions on which factors contribute to the efficacy-effectiveness gap. The absence of post-marketing studies is remarkable, but might be explained by the rapid development of new drugs for each indication, making post-marketing studies on ‘old’ drugs less interesting to be performed. Because data in international cancer databases were not sufficiently accurate, these data were not suitable to be included in our study. The lack of data on treatment outcomes does, however, underline the need for more complete databases.

The 3 month cutoff that was chosen for determination of a clinically relevant change in OS might seem arbitrary, but was derived from the NICE ‘guideline on appraising life-extending, end of life treatments’ (for patients with a life expectancy < 24 months). Since the majority of the included OMPs in our study was authorized for advanced, progressive or metastasized cancers, we believe this cutoff is justified.

## Conclusion

Forty percent of the authorized oncologic OMPs did not show a clinically relevant gain in OS in the real-world setting, suggesting that an efficacy-effectiveness gap exists for oncologic OMPs. Our study suggests that the type of marketing authorization, type of primary study endpoint, and study power all contribute to the efficacy-effectiveness gap to a certain extent. All OMPs with high ESMO-MCBS scores showed a good real-world effectiveness, indicating that this scoring system may be of high value in the assessment of drug dossiers as well as reimbursement decisions. Furthermore, post-marketing surveillance of OMPs with a conditional/exceptional marketing authorization in particular is important to monitor the real-world effectiveness.

## Additional files

Additional file 1: EPAR comments. (DOC 50 kb)

Additional file 2: Overview of pivotal studies and post-marketing studies. (DOC 379 kb)

## Abbreviations

COMPASS: Clinical evidence of Orphan Medicinal Products - an ASSessment tool
DFS: Disease-Free Survival
ECOG: Eastern Cooperative Oncology Group
EMA: European Medicines Agency
EPAR: European Public Assessment Report
ESMO-MCBS: European Society for Medical Oncology Magnitude of Clinical Benefit Scale
EU: European Union
FDA: Food and Drug Administration
HTA: Health Technology Assessment
NICE: National Institute for Health and Care Excellence
OMP: Orphan Medicinal Product
ORR: Objective Response Rate
OS: Overall Survival
PFS: Progression-Free Survival
PRIME: PRIority Medicines
QoL: Quality of Life
RFS: Recurrence-Free Survival
TTF: Time to Treatment Failure
TTP: Time To Progression
