# Supplementary material for: Oncologic orphan drugs approved in the EU – do clinical trial data correspond with real-world effectiveness?
Source: Orphanet J Rare Dis. 2018 Nov 28;13:214. doi: 10.1186/s13023-018-0900-9 (PMC6263065; doi:10.1186/s13023-018-0900-9)
Supplement: Supplementary file 1 — EPAR comments. (DOC 50 kb) [file 13023_2018_900_MOESM1_ESM.doc]

| *Additional file 1: EPAR comments** | | |
| --- | --- | --- |
| **Drug** | **Indication** | **Comments in EPAR** |
| Imatinib | GIST | In one of the pivotal studies, the primary endpoint (OS) was changed to RFS on the basis of discussions with Cancer Therapy Evaluation Program and the US Food and Drug Administration 6 months before the first planned efficacy interim analysis. RFS benefit is mainly limited to the high risk patient groups. More data are needed in moderate risk tumors, and no benefit is apparent in the low risk group. |
| Imatinib | DFSP | The MAH provides limited data on the antitumor effect of imatinib in patients with DFSP, due to the extreme rarity of this disease. Nevertheless, the biological plausibility is undisputable. The MAH should commit to provide yearly updated information on ongoing clinical trials of imatinib in DFSP. |
| Mitotane | Adrenocortical carcinoma | This ‘well-established use’ application consists of published studies of poor quality. Most of them are retrospective case-series with mitotane being evaluated as palliative therapy in advanced stages or as adjuvant treatment to surgery in extremely heterogeneous population in terms of disease stage, objective of the therapeutic intervention (adjuvant to surgery or palliative treatment in non-surgical population), associated treatments, and dose regimen. The CHMP considered that the indication should be restricted to unresectable adrenal carcinoma. The published data do not provide sufficient evidence to support any effect of mitotane on survival. |
| Sunitinib | GIST | Trial was unblinded early when a planned interim analysis showed significantly longer time to tumor progression with sunitinib. |
| Sunitinib | RCC | None |
| Sorafenib | RCC | It was considered unfortunate that the blind was broken and that patients were crossed-over based on PFS data, as survival results were expected to be either immature or highly confounded by crossover to active therapy. However, after consultation of the oncology SAG, the CHMP considered that PFS per se could be considered as a measure of the clinical benefit of sorafenib. Based on PFS data, it was concluded that a favorable and clinically meaningful effect has been demonstrated. |
| Sorafenib | HCC | The exclusion of patients with liver impairment > Child-Pugh A was accepted due to seemingly less favorable results in Child-Pugh B patients in an exploratory, single arm study. There are two alternative primary endpoints (i.e. efficacy established if either positive), OS and TTSP. The latter was introduced based on recommendations from FDA. However, at the time of the CHMP advice, only survival was discussed (and endorsed).The first interim analysis was conducted on too immature data. |
| Sorafenib | Thyroid carcinoma | None |
| Temsirolimus | RCC | None |
| Trabectedin | STS | There were a number of methodological deficiencies that hampered the assessment of the efficacy of trabectedin in the treatment of STS. Crucial aspects included the timing and sponsor’s access to data at the time of protocol amendment leading to a change of the primary endpoint. |
| Trabectedin | Ovarian cancer | The absolute magnitude of PFS prolongation (about 1.5 months) is not impressive considering that there is no clear support of OS prolongation. Whereas PFS may be a valid surrogate for the OS endpoint in first-line treatment (chemo-naïve patients) this correlation may not be well substantiated in later-line therapy situations. |
| Mifamurtide | Osteosarcoma | CHMP found that there were a number of unexplained findings and uncertainties about the treatment effect associated with mifamurtide. The treatment effect was different according to associated chemotherapy and appeared to be present only when mifamurtide is used in conjunction with ifosfamide. However, the SAG considered that the unexplained observations and uncertainties are well within the range of what is observed with other cancer products. The SAG agreed that based on the clinical efficacy data presented, treatment with MTP was associated with clinically significant benefits in the proposed indication. There are not sufficient data to recommend the use of mifamurtide in patients >30 years of age. |
| Everolimus | RCC | The trial was terminated early because the predefined stop criteria were met. The option to crossover from placebo probably confounds the survival result and prevents the detection of a significant difference in OS. The clinical relevance of PFS as the primary endpoint and the benefit of approximately 3 months difference in PFS between treatments were discussed, especially since this was in disagreement with scientific advice from the SAWP, which advocated the use of OS as the preferred endpoint. Moreover, the significance of the primary endpoint was questioned, in particular since there is no additional support for a patient benefit in terms of QoL and PROM data presented. |
| Cabozantinib | Medullary thyroid cancer | Based on the results of the pivotal study where 75% of patients required at least one dose reduction after steady-state concentrations were reached and 41% of patients needed two dose reductions due to persistent toxicity, serious concerns were raised regarding the appropriateness of the dose proposed for registration. Based on efficacy data alone, it is not clear whether a lower dose than the MTD could be equally effective. |
| Olaparib | Ovarian cancer | Patients with stable disease following platinum-based chemotherapy were not included in the pivotal trial. Additional studies are needed to support efficacy of olaparib for maintenance in patients with stable disease following platinum-based chemotherapy. |
| Ramucirumab | Gastric cancer | None |
| Dinutuximab | Neuroblastoma | The trial was stopped prematurely because superiority of immunotherapy over the control had been established on EFS. However, this was not justified as it is obvious that the stopping boundary was not crossed. EFS results have to be interpreted with caution and the OS results are considered paramount to evaluate the treatment benefit. |
| Lenvatinib | Thyroid cancer | None |
| Olaratumab | STS | None |
| Liposomal Irinotecan | Pancreatic cancer | The study population was heterogeneous, mainly in terms of prior lines of therapy and therefore also in terms of exposure to different cytotoxic agents. Whilst not considered to be the most informative, the study design, including 5FU/LV alone as reference therapy, was accepted from a regulatory perspective. The number of different 5FU/LV regimens still in clinical use is large, meaning that there is no “gold standard”. |

*5-FU= 5 fluorouracil; CHMP= Committee for Medicinal Products for Human Use; DFSP= dermatofibrosarcoma protuberans; EPAR= European public assessment report; FDA= food and drug administration; HCC= hepatocellular carcinoma; LV= leucovorin; MAH= marketing authorization holder; MTD= maximum tolerated dose; OS= overall survival; PFS= progression-free survival; pNET= pancreatic neuro-endocrine tumor; PROM= patient reported outcome measure; QoL= quality of life; RCC= renal cell carcinoma; SAG= scientific advisory group; SAWP= scientific advice working party; STS= soft tissue sarcoma; TTSP= time to symptomatic progression

*Comments are literally copied from EPAR documents:* [*http://www.ema.europa.eu/ema/index.jsp?curl=pages/medicines/landing/epar_search.jsp&mid=WC0b01ac058001d124*](http://www.ema.europa.eu/ema/index.jsp?curl=pages/medicines/landing/epar_search.jsp&mid=WC0b01ac058001d124)
