# Supplementary material for: Oncologic orphan drugs approved in the EU – do clinical trial data correspond with real-world effectiveness?
Source: Orphanet J Rare Dis. 2018 Nov 28;13:214. doi: 10.1186/s13023-018-0900-9 (PMC6263065; doi:10.1186/s13023-018-0900-9)
Supplement: Supplementary file 2 — Overview of pivotal studies and post-marketing studies. (DOC 379 kb) [file 13023_2018_900_MOESM2_ESM.doc]

| *Additional file 2: Overview of pivotal studies and post-marketing studies* | | | | |
| --- | --- | --- | --- | --- |
| **Drug** | **Indication** | **Therapeutic evidence of pivotal study** | **Main results of post-marketing studies** | **OS of standard of care in literature (historical)** |
| Imatinib | GIST | One open-label randomized trial (n=147, unresectable or metastatic GIST) in patients with advanced GIST. Primary endpoint: ORR. No patient had a CR, 53.7% of the patients had PR. One randomized double-blind placebo-controlled trial (n=713, localized, primary GIST) in adjuvant setting. Primary endpoint RFS. 1-year RFS was 98% in imatinib group versus 83% in placebo group, with an overall HR of 0.35 (0.22–0.53; p<0.0001). OS was not reached. | Eight post-marketing studies, including 3 randomized open-label studies [1-3]. No double-blind studies performed. **OS rates varied from 85-92% (1-yr)** [3, 4]**, 69-94% (2-yr)** [1-6] **and 54-74% (3-yr)** [4, 7]. In a single-arm study, **median OS was 41.1 months** [8]. In 1 study, continuation vs cessation were compared in patients with >3 year imatinib use: 2-yr PFS was 80% vs 16% respectively, but OS in both groups was similar [2]. A retrospective analysis found that grade ≥ 3 adverse events occurred in 70% of the patients [6]. No data on QoL. | Median OS was **22 months** for patients with metastatic GIST and 9-12 months for patients with local recurrence [9].  Median OS was **9–19 months** in the pre-imatinib era [1]. |
| Imatinib | Dermatofibrosarcoma protuberans | One open-label single-arm study (n=12, refractory to standard therapy or in absence of conventional therapy) with biomarker: ORR. 4 patients had CR (33%), 6 patients had PR (50%). Median time to progression was 23.9 months. OS was not analyzed. | Four post-marketing studies, of which 3 retrospective studies and 1 prospective. Two studies included OS-analysis, with **1-year OS rates between 85-96% and 3-year OS rates between 60-77%** [10, 11]. No data on QoL. | 3-year OS rate 66% (for soft tissue sarcoma, not specified).[12] |
| Mitotane | Adrenocortical carcinoma | Evidence based on a literature analysis with mainly series of case reports describing effects on mortality, remission and tumor size. The published data do not provide sufficient evidence to support any effect of mitotane on OS. | No post-marketing studies  (Two case reports, both showing long-lasting disease-free survival of up to 10 years [13, 14].) | Median survival in patients with **unresectable tumors 3-9 months**. After complete resection **median survival 13-28 months** [15]. |
| Sunitinib | GIST | One adequate placebo-controlled trial; n=312 (advanced GIST, resistant or intolerant to imatinib). Endpoint: TTP (27.3 vs 6.4 weeks). Trial was unblinded early when a planned interim analysis showed significantly longer time to tumor progression with sunitinib. Median OS was not reached. | Seven post-marketing studies, only 1 placebo-controlled randomized trial in which statistical analysis showed that OS converged in sunitinib and placebo arms (103/118 placebo patients crossed over to open-label sunitinib) (median 72.7 vs 64.9 weeks; HR 0.876; p=0.306) [16]. **Median OS** in remaining studies ranged from **14.1 – 17.6 months** [17-20]. No QoL-related measures. | Median OS was **22 months** for patients with metastatic GIST and 9-12 months for patients with local recurrence [9]. Median OS was **9–19 months** in the pre-imatinib era [1]. |
| Sunitinib | RCC | Only interim results of an uncontrolled trial (n=106, metastatic RCC, 2^nd^ line) at time of authorization (conditional approval). Post-marketing obligation study (n=750, metastatic RCC) compared sunitinib with IFN-α and showed a longer PFS (11 vs 5 months), higher ORR (31% vs 6%) and a better QoL in sunitinib group. In both studies, OS was not reached and could not be calculated. | Four post-marketing studies, of which 2 expanded access/compassionate use trials. **Median OS ranged from 18.2 – 27.2 months** [21-23]. In elderly patients median **OS was 18.3 months** [24]. No data on QoL. | OS following progression after cytokine therapy is approximately **10-13 months** [25]. |
| Sorafenib | RCC | Adequately sized randomized double-blind placebo-controlled trial (n=903, unresectable and/or metastatic RCC) with FPS as primary endpoint: 5.5 months in sorafenib group vs 2.8 months in the placebo group (p<0.01). Interim analysis of OS showed that sorafenib reduced the risk of death, but this was not statistically significant. | Two open-label randomized trials and two extensions of pivotal study. In final OS analysis, when post–crossover placebo survival data were censored, the OS difference was **17.8** vs 14.3 months, in favor of sorafenib. Sorafenib was compared to tivozanib and axitinib. A trend toward longer **survival** on sorafenib compared to tivozanib was seen (median OS **29.3** vs 28.8 months) [26]. In another study, median OS was 20.1 months (95% CI 16.7–23.4) with axitinib and **19.2 months** (17.5–22.3) with sorafenib [27, 28]. In comparison to tivozanib and axitinib, no differences were seen in QoL. | Metastatic RCC: median survival time **6-12 months**, **2-year survival rate of 10-20%** [29]. OS following progression after cytokine therapy is approximately **10-13 months** [25]. |
| Sorafenib | HCC | Randomized double-blind placebo-controlled trial (n=602, advanced HCC) with adequate endpoints: OS and TTSP. Median OS was significantly longer in sorafenib group than in placebo group. | Eleven post-marketing studies, of which 2 retrospective studies. Ten studies included patients with advanced HCC, only one study included patients with HCC suitable for curative treatment [30]. In prospective studies, the **median OS ranged from 5-10.2 months** [31-37]. In one study, **1-year OS rate was 31.7% and 2-year OS rate was 18.2%** [35]. Randomized trials showed that OS did not differ significantly when comparing sorafenib to linifanib or brivanib [32, 36]. A retrospective study showed no OS difference between sorafenib and transarterial chemoembolization [38]. Sorafenib showed a significant difference in OS when compared to sunitinib, capecitabine and placebo [31, 33, 34]. One open-label study showed mixed results on QoL (significant effect from baseline in only some subscales). | 5-year survival rates of up to 60-70% in well-selected patients with *early stage* HCC (after surgery/ablation) [39]. **Median OS of untreated patients with non-resectable HCC is <6 months** [40]. |
| Sorafenib | Thyroid carcinoma | Randomized double-blind placebo-controlled trial (n=417), primary endpoint: PFS. PFS was two times longer in sorafenib group vs placebo group. However, ORR was low in both groups (sorafenib: 12.2%) and OS was not reached. | Three post-marketing studies, of which 2 retrospective studies and 1 off-label use study. **Median OS ranged from 28.38 to 56 months** [41, 42]. No QoL data. | Median survival for patients with radioactive iodine (RAI)-refractory differentiated thyroid cancer (DTC) and distant metastases is **2.5-3.5 years** [43, 44]. |
| Temsirolimus | RCC | Randomized open-label study (n=626) in previously untreated patients: IFN-a vs temsirolimus. Primary endpoint (OS) was significantly longer in temsirolimus group. | Five post-marketing studies; two retrospective analyses, two non-randomized trials and one randomized trial. Temsirolimus was either used as 1^st^, 2^nd^ or 3^rd^ line treatment. **Median OS was between 11.6-18 months** [45-47]. One randomized trial comparing temsirolimus and sorafenib found a significant OS difference in favor of sorafenib [45]. No QoL data. | Treatment with IL-2 and/or IFN-a results in **median OS** **of 12-17.5 months** [48]. Metastatic RCC: median survival time **6-12 months**, **2-year survival rate of 10-20%** [29]. |
| Trabectedin | STS | One phase II randomized open-label study with 2 dosing schedules of trabectedin, after failure of first-line chemotherapy. Endpoint: TTP (n=270, advanced or metastatic STS). Trial demonstrates superior disease control with the q3 weeks 24-hour trabectedin regimen in liposarcomas and leiomyosarcomas. No significant differences were shown in OS. | Three post-marketing studies. **Median survival time ranged from 11.9 months** (open-label single arm, n=807) [49] **to 19.3 months** (retrospective analysis, n=25) [50]. In a randomized open-label, active-controlled study (n=509), results of OS were highly censored but favored the trabectedin group (median OS of trabectedin versus dacarbazine, **12.4 months** vs 12.9 months) [51]. No data on QoL. | **Median survival: 8-13 months** from initiation of first-line chemo (anthracyclines and ifosfamide) and **6 months** after failure of standard treatment [52].   Response percentage with first line therapy (doxorubicin or ifosfamide) is 20-30%. **Median survival is about 1 year** [53]. |
| Trabectedin | Ovarian cancer | Open-label randomized study that compares trabectedin plus PLD with PLD alone after platinum-based chemotherapy. Endpoint: PFS. Small but statistically significant difference of PFS in favor of trabectedin+PLD. ORR was significantly better in trabectedin+PLD group in platinum-sensitive patients. No significant differences between treatment arms in QoL. | Two retrospective analyses and two extensions of pivotal study. **Median OS was 16.3 months** (platinum-sensitive patients, retrospective analysis, n=34) [54]. Another retrospective analysis of ‘real-life efficacy’ showed median **OS of** **17.6 months** [55]. An extension of the pivotal study showed an **overall median OS of 22.2 months** in trabectedin+PLD group vs 18.9 months in PLD group with a HR which was *not* statistically significant [56]. Addition of trabectedin to PLD led to little or no decrement in patient-reported functional status and symptoms [57]. No data on QoL. | Chemosensitive disease: **median OS > 30 months**.  Chemoresistant disease: **median OS > 8 months** [58]. |
| Mifamurtide | Osteosarcoma | One prospective randomized trial (n=677, non-metastatic). Primary endpoint: EFS. All patients received identical doses of cisplatin, doxorubicin, and high-dose methotrexate and underwent definitive surgical resection of the primary tumor. Patients were then randomly assigned to receive or not to receive ifosfamide and/or mifamurtide. The addition of mifamurtide to chemotherapy might improve EFS, but additional studies are necessary to explain the interaction between ifosfamide and mifamurtide. | No post-marketing studies. | No therapy: 5-year survival rate 15%, **(neo)adjuvant chemotherapy: 5-year survival rate 65%** [59]. |
| Everolimus | RCC | Randomized double-blind placebo-controlled trial (n=416, metastatic RCC and progressed after sunitinib and/or sorafenib). The study was stopped early because the predefined stop criteria were met. Median PFS (primary endpoint) was significantly longer in everolimus group vs placebo group. OS was not reached. No difference between groups in PROMs. | Eight post-marketing studies, of which 4 randomized trials and one study with updated results of the pivotal study. **Median OS ranged from 14.8-32 months** [60-63]. When compared to cabozantinib and nivolumab, median OS was significantly worse with everolimus [60, 62]. The rate of QoL decrease in everolimus was significantly greater than in placebo arm [64], whereas in another study QoL improvement with nivolumab was significantly better than with everolimus [62]. | Local RCC: 5-year survival rates of 89.6% Advanced/metastatic RCC: 5-year survival rates of 9.5% [65]. |
| Cabozantinib | Medullary thyroid cancer | Randomized double-blind placebo-controlled trial (n=330, metastatic medullary thyroid cancer). Endpoint: PFS. Median PFS was 11.2 months for cabozantinib versus 4.0 months for placebo (HR 0.28, p=0.001). One-year OS was also significantly better in cabozantinib group (47% vs 7% in placebo). | No post-marketing studies. | The 10-year OS rate is estimated at 40% or less in patients with locally advanced or metastatic disease [66]. |
| Olaparib | Ovarian neoplasms | Randomized double-blind placebo-controlled study (n=265, platinum-sensitive relapsed). Primary endpoint (PFS) was significantly better in olaparib group. OS was not reached, but showed no difference in interim analysis. No significant between-group differences in disease-related symptoms or health-related QoL. | No post-marketing studies. | 5-year survival rate 44% across all stages and 27% for advanced stages [67].  **5-year OS is 44% for BRCA1 carriers, 52% for BRCA2 carriers** [68]. |
| Ramucirumab | Gastric cancer | Two phase III randomized placebo-controlled trials (n=355 and n=665), with OS as primary endpoint. One study shows that median OS is significantly longer with ramucirumab + paclitaxel than with placebo + paclitaxel. The other study shows that OS is significantly longer with ramucirumab monotherapy when compared to placebo. QoL did not improve with ramucirumab compared with placebo. | No post-marketing studies. | Median OS for patients with advanced gastric cancer is approximately 1 year [69]. 5-year survival rate is 4.2 % for patients with metastatic disease [70]. |
| Dinutuximab | Neuroblastoma | Open-label randomized controlled trial (n=230), endpoint: EFS. Trial stopped prematurely because superiority of immunotherapy over control was established on EFS. Estimates for 2-year EFS were 66.3% vs. 46.4% for the immunotherapy and control arms, respectively. OS results demonstrate benefit of immunotherapy, with a survival benefit of 14% at 3 years (82% vs 68%; p<0.02) and an HR of 0.57 (0.36;0.89). | No post-marketing studies. | EFS rates are 30 to 40% for children with high-risk neuroblastoma [71]. |
| Lenvatinib | Thyroid cancer | Randomized double-blind placebo-controlled trial (n=392, progressive thyroid cancer refractory to iodine-131). Endpoint: PFS. Median PFS was significantly longer in lenvatinib vs placebo (HR for progression or death, 0.21; 99% CI, 0.14 to 0.31; p < 0.001). No significant difference in OS between the groups. | No post-marketing studies. | Median survival for patients with RAI-refractory DTC and distant metastases is 2.5-3.5 years [43, 44]. |
| Olaratumab | STS | One randomized phase Ib/II open-label study, comparing olaratumab+doxorubicin with doxorubicin alone (n=133). Primary endpoint PFS showed no significant differences. Secondary endpoint OS, however, showed a significant difference: 26.5 months (20.9–31.7) with olaratumab plus doxorubicin and 14.7 months (9.2–17.1) with doxorubicin (HR 0.46, 0.30–0.71, p = 0.0003). | No post-marketing studies. | Median survival time in patients with metastatic STS is 11 to 15 months [72]. |
| Nanoliposomal irinotecan | Pancreatic cancer | Phase III, randomized, open-label trial in patients with metastatic pancreatic cancer after previous gemcitabine-based therapy (n=417). Three groups; a) nanoliposomal irinotecan, b) 5-FU and LV, c) nanoliposomal irinotecan with 5-FU and LV. Primary endpoint (median OS) in patients with nanoliposomal irinotecan plus 5-FU and LV was 6.1 months vs 4.2 months with 5-FU and LV (p = 0.012). Median OS did not differ between nanoliposomal irinotecan monotherapy group and 5-FU and LV group. | No post-marketing studies. | Median OS in patients with advanced disease is less than one year [73]. |

5-FU= 5-Fluorouracil; CR= complete response; DTC= differentiated thyroid cancer; EFS= event-free survival; GIST= gastrointestinal stromal tumor; HCC= hepatocellular carcinoma; HR= hazard ratio; LV= leucovorin/ folinic acid; ORR= objective response rate; OS= overall survival; PFS= progression-free survival; PLD= pegylated liposomal doxorubicin; pNET= pancreatic neuroendocrine tumor; PR= partial response; PROM= patient reported outcome measure; QoL= quality of life; RAI= radioactive iodine; RCC= renal cell carcinoma; RFS= recurrence-free survival; RPSFT= rank preserving structural failure time; STS= soft tissue sarcoma; TC= thyroid cancer; TTP= time to progression; TTSP= time to symptom progression

**References**
